# Multi-Drug Resistance Tuberculosis (MDR-TB) Challenges in India: A Review

**DOI:** 10.7759/cureus.50222

**Published:** 2023-12-09

**Authors:** Deepak Vishwakarma, Abhay Gaidhane, Sweta Sahu, Ashwini S Rathod

**Affiliations:** 1 School of Epidemiology and Public Health, Datta Meghe Institute of Medical Sciences, Wardha, IND

**Keywords:** mdr, india, challenges, multi-drug resistant tb, tb – tuberculosis

## Abstract

Tuberculosis (TB) is an increasing public health concern in India. The development of resistance to two of the most effective medications, rifampicin, and isoniazid, is the largest worldwide challenge in the management of TB. An epidemiological indicator used to evaluate the effectiveness of the TB management program is the initial medication resistance level. Our search for published papers in English using Medical Subject Heading phrases was conducted through PubMed, Google Scholar, and Scopus. We also looked through several official sources to learn the most recent information on multi-drug resistance in India. Multi-drug resistance tuberculosis is a significant risk to human health globally. Stigmatization or discrimination of those who have TB or who are impacted by it can make the disease more difficult to manage medically and socially, and it is ultimately responsible for missed opportunities for diagnosis and treatment, interruptions of care, and unsuccessful outcomes. Eliminating TB is complicated by stigma.

## Introduction and background

Microorganisms that cause tuberculosis (TB) are airborne infections that spread from person to person. Although TB mostly impacts not only the lungs but also the spine, brain, kidneys, and other body organs. Most of the time TB is curable; however, if treatment is not received, TB patients may not survive. whenever a person who has TB illness of the throat or lungs speaks, sings, coughs, or sneezes. Neighbors may inhale these microorganisms and acquire an infection [1].

TB bacteria resistant to fluoroquinolones, rifampin, and isoniazid are named as causing pre-extensively drug-resistant tuberculosis (pre-XDR TB) [2]. Since isoniazid and rifampicin are the most effective first-line TB medications, missing doses of these medications can cause multi-drug resistance tuberculosis (MDR-TB) to arise [3]. Since the beginning of anti-tubercular chemotherapy, there has been evidence of drug-resistant tuberculosis (DR-TB). However, more recently, MDR-TB has become a source of increasing concern and is posing a danger to efforts being made worldwide to combat the disease. Treating MDR-TB is a tough, costly, and frequently unsuccessful treatment. The challenges with MDR-TB are the main topic of this article [4].

In 2022, an estimated 410,000 persons (95% UI: 370,000-450,000) will have developed multi-drug resistance or rifampicin resistance tuberculosis (MDR/RR-TB). A significantly smaller number of patients received a diagnosis and began treatment: 175,650 in 2022, roughly two out of every five individuals in need remains below the pre-pandemic figure of 181,533 in 2019 [5]. Additionally, isoniazid resistance, which has overall resistance rates of 16% and varied percentages among newly diagnosed and previously treated patients, contributes to rifampicin-resistant tuberculosis (RR-TB) [6].

MDR-TB is a significant task for TB control in various contexts, although the exact worldwide load is still unclear. Reliable assessments of MDR-TB frequency are critical at the global and country levels to strategize and enhance DR-TB management within national TB control programs. Fortunately, treatment for MDR-TB has proven feasible and successful even in low-resource settings [7]. Over the years, India has seen a rising number of people diagnosed with MDR-TB. However, due to a lack of an extensive monitoring system and trustworthy prevalence studies, it is still difficult to ascertain the true scope of MDR-TB in the nation. To address this, In India, every tertiary care center will have its primary and acquired resistance levels evaluated as part of our initiative [8].

## Review

Methodology

**Figure 1:**
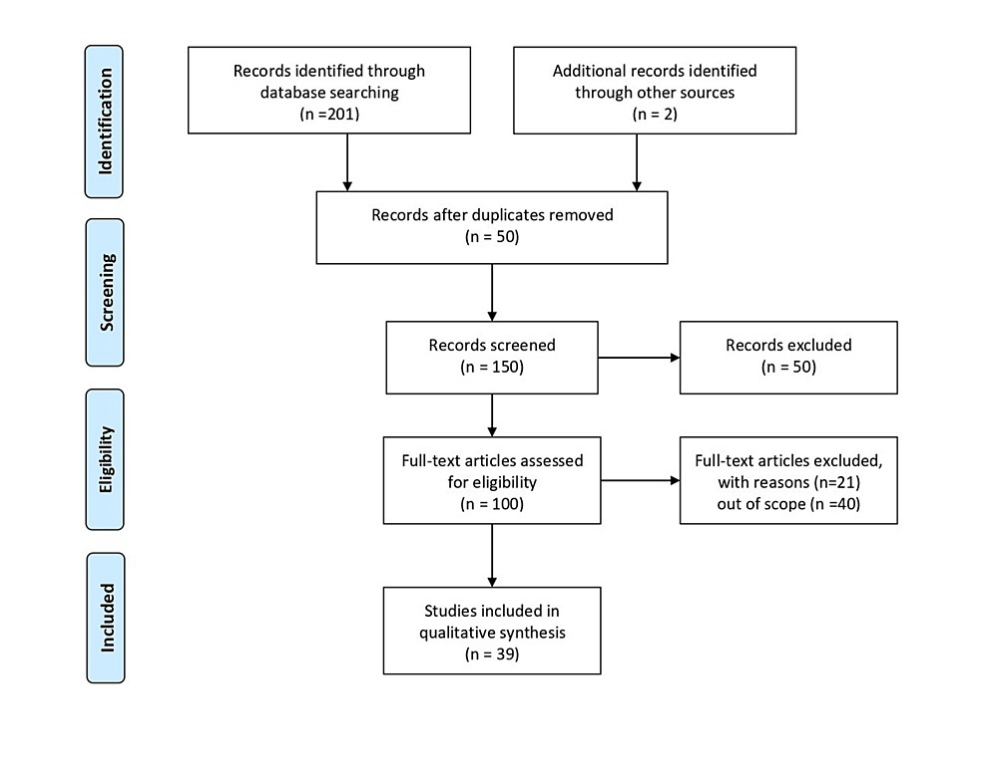
PRISMA flow diagram depicting how the studies were selected PRISMA: Preferred Reporting Items for Systematic Reviews and Meta-Analyses

We searched PubMed, Scopus, and Google Scholar using Medical Subject Heading terms like "Multi-Drug Resistance Tuberculosis" "MDR" "Challenges" "In India" and Boolean operators "AND/OR" to find published papers, studies, and research in the English language. We incorporated several studies on MDR-TB in India. The inclusion criteria were based on publications published between the years 1970 and 2023 that only addressed the challenges posed by MDR-TB in India. The selection process for the studies is shown in Figure 1 below.

Definition

Isoniazid and rifampin, two of the most potent and commonly used TB medications, are just two of the drugs that cause MDR-TB. Currently, the drug is included in routine treatment for all TB patients [9].

Multi-drug resistance tuberculosis in India: challenges and burden

As one of the highest rates of MDR-TB in the world, TB with MDR-TB is a severe issue in India. According to the World Health Organization (WHO), India recorded 64,000 MDR-TB infections in 2012, making it the nation with the greatest prevalence globally and responsible for 22% of all MDR-TB cases worldwide [10]. Three hundred thousand cases of pulmonary TB were recorded in India in 2013, which is believed to have had 64,000 MDR-TB patients [10].

The incidence of new infections of MDR-TB in 2013 was approximately 480,000 globally, and about 190,000 cases resulted in fatalities [10]. Since the inception of anti-TB medications, DR-TB has been a recurrent issue in India. Initially, it was believed that DR-TB was not easily transmitted, and most cases were attributed to improper anti-TB medication use [11]. Efforts to prevent resistance have seen high-quality direct focuses on directly observed treatment short-course (DOTS) programs that monitor drug compliance to avoid resistance formation. However, it was later discovered that drug-resistant bacteria were still contagious, with similar infection risks for drug-sensitive and treatment-resistant bacilli in individuals who came into contact with untreated patients.

DR-TB remains one of the primary obstacles to India's progress toward TB elimination [12]. An estimated 130,000 cases of DR-TB were reported in India in 2018. However, only 44% of suspected MDR cases (approximately 58,347 cases) were diagnosed in that year, and about 35.8% of them (46,569 patients) received treatment [13]. In the same year, around 2.8% of new MDR/RR-TB cases were reported, and the predicted percentage of MDR/RR-TB cases with prior treatment was 12%. About 135,000 MDR/RR-TB cases were reported, with 32% of new cases being notified and 82% of previously treated patients testing positive for rifampicin resistance. Additionally, 26,832 MDR/RR-TB cases had their second-line medication resistance tested [14]. Testing on line probe assays (LPA) in Table 1.

**Table 1:** Testing on line probe assays (LPA) FLQ: Fluoroquinolones; SLI: Second-line injectable drug Reference: [12-14]

State	First-line line probe assay				Second-line line probe assay			
	Samples tested	Resistant to both isoniazid and rifampicin	Resistant to rifampicin	Resistant to isoniazid	Samples tested	Resistant to both FLQ and SLI	Resistant to FLQ	Resistant to SLI
Himachal Pradesh	6258	108	42	229	407	3	35	0
Gujarat	9862	357	109	608	3780	131	1005	82
Delhi	22530	1812	306	1496	3233	77	1025	63
Maharashtra	47018	5947	1839	3293	11538	865	4038	115
Jammu and Kashmir	2316	33	11	88	155	6	21	0
Sikkim	325	39	44	49	154	2	36	8
Karnataka	29042	854	487	2105	3613	43	505	36
Kerala	4809	121	174	310	407	8	29	6
Punjab	2753	132	33	110	496	5	120	1
Rajasthan	12027	1080	703	991	3733	82	821	44
Andhra Pradesh	19414	530	211	1439	2079	5	211	10
Bihar	11746	2017	257	611	3309	165	1132	29
Tamil Nadu	17859	566	371	1346	2192	15	262	13
Assam	2869	206	41	179	425	6	101	8
Jharkhand	4639	212	143	193	448	14	107	2
Nagaland	345	21	5	32	30	0	2	0
Meghalaya	976	107	7	70	127	3	31	3
Telangana	19593	541	268	1108	1929	16	202	7
Chhattisgarh	5156	120	40	320	531	4	76	5
Haryana	5823	155	162	389	820	14	99	18
Madhya Pradesh	22312	1012	470	1418	3336	54	868	10
Odisha	6061	151	25	156	487	5	59	4

Discussion

India is considered a hotspot for *Mycobacterium tuberculosis* infection, and together with Russia and China, accounts for about 62% of the global MDR-TB burden [6,13]. Despite major improvements in the management and treatment of TB, especially in developing countries, TB remains a serious public health problem. About 30% of the world's TB burden falls on India alone [15].

The DOTS method, employing first-line medicines in standardized short-course chemotherapy (SCC), is currently the primary approach for TB control worldwide. However, DOTS treatment may not offer sufficient cure rates for MDR-TB patients [16]. Treatment with anti-TB medications becomes essential to address this, as traditional TB control strategies like the Bacillus Calmette-Guerin vaccine and chemoprophylaxis appear ineffective. The rise in DR-TB cases significantly threatens successful disease treatment [17].

The current threat comes from *M. tuberculosis* strains that appear more resistant to the more potent bactericidal anti-TB drugs, such as rifampicin and isoniazid, often employed in TB control programs. Despite the early discovery of drug resistance during the use of streptomycin [17], concerns about the rise of bacteria that are resistant to antibiotics and the effectiveness of chemotherapy in treating these infections persist [18].

The issue of DR-TB is primarily an artificial problem, emphasizing the importance of prescribing appropriate treatment regimens for specified durations. Understanding the drug resistance pattern in specific areas is crucial to developing effective treatment plans, as it varies across different regions and time periods [19]. In efforts to combat MDR-TB, it has been recommended that nations with effective DOTS programs and endemic populations consider supplemental facilities, such as the DOTS-Plus program, to identify and treat MDR-TB patients [20].

Types of Drug Resistance and Management Strategies in Tuberculosis

Drug resistance in TB falls into two categories: acquired and primary. Primary drug resistance is used to describe drug-resistant instances in people who have never used anti-tubercular therapy before. Conversely, people who have already had chemotherapy develop acquired drug resistance [21]. The term "acquired drug resistance" refers to the resistance that manifests in a patient who has previously received chemotherapy [15].

Multi-Drug Resistance Tuberculosis

TB bacteria can acquire resistance to the antimicrobial medications used to treat the condition. When TB does not react to a minimum of isoniazid and rifampicin, the two most potent anti-TB medications, it is referred to as MDR-TB. Person-to-person transmission and improper administration of TB treatment are the two causes of the ongoing emergence and spread of multi-drug resistance. Most TB patients are treated by a rigorously adhered-to, six-month treatment regimen that is given to patients with assistance and monitoring [22].

Pre Extensively Drug-Resistant Tuberculosis

Pre-XDR TB is a relatively new term that refers to TB that is resistant to rifampicin and isoniazid, as well as one or both of the following additional drugs: a fluoroquinolone or an injectable second-line agent, but not both of these drugs simultaneously. Therefore, under the usual MDR-TB regimen, pre-XDR TB subjects with fluoroquinolones or isoliquiritigenin resistance receive less effective medicines. It might intensify existing drug resistance even more and speed up the development of extensively drug-resistant tuberculosis (XDR-TB) [23].

Extensively Drug-Resistant Tuberculosis

Widespread DR-TB, which is resistant to isoniazid, rifampin, and kanamycin, a rare MDR-TB disease, is caused by TB bacteria and other second-line injectable antibiotics as well as amikacin, capreomycin, and kanamycin. Few effective treatments exist for XDR-TB because it poses a greater risk, and individuals with compromised immune systems, like HIV, are more susceptible to its harmful consequences. XDR-TB is especially dangerous for persons who have HIV or other illnesses that impair the immune system. Once infected, these individuals have a higher likelihood of contracting TB disease, which increases their risk of dying from TB.

Source and Causes of MDR

MDR-TB occurs as a result of random and unusual chromosomal changes to *M. tuberculosis* that make the bacteria less susceptible to certain drugs [24]. Drug resistance can occur for a number of reasons, including changes in drug targets, activation of efflux pumps on the bacterial surface, disruption of drug activation, and production of drug-deactivating enzymes [25]. Because the mutation rate for isoniazid is 10-5, it is 10-7 for rifampicin [26]. Drug resistance can occur in two ways, due to which the frequency of MDR-TB is low. Primary resistance will develop in patients who come into contact with an individual who already has a drug-resistant strain [27]. Secondary or acquired resistance emerges as a result of poor management, healthcare providers, and patient-related factors [28]. They involve (i) inadequate or failing TB control programs, which lead to inadequate provision of suitable care [29]. (ii) inadequate case management, the use of subpar medications, an inadequate or inconsistent drug supply, and a lack of monitoring; (iii) healthcare workers' lack of knowledge of epidemiology, treatment, and control [30]. (iv) incorrect regimens are given; (v) chemotherapy is discontinued due to side effects; (vi) patients don't stick to the prescribed drug therapy; and (vii) anti-TB drugs are marketed over the counter without a prescription [31]. (viii) high bacillary load, (ix) patient illiteracy and low socioeconomic (x) status, the HIV infection epidemic, (xi) laboratory delays in isolating and testing *M. tuberculosis* separate, (xii) the use of unstandardized laboratory techniques, poor-quality drug powders, and a lack of adequate measures, and (xiii) the use of anti-TB drugs for conditions other than TB [32].

Diagnosis of Multi-Drug Resistance

There are essentially two ways to diagnose MDR-TB: first, by phenotyping drug susceptibility testing (DST), which involves culturing the isolate on liquid or solid media containing a specific antibiotic, or by molecular detection of drug-resistance gene mutations. The latter can even be given automatically at the point of patient care. The second is the GeneXpert *M. tuberculosis*/resistance to rifampicin (Mtb/RIF) test, the primary TB diagnostic tool in many endemic areas. Examples of forward molecular diagnostics include array-based platforms and LPA [33].

Molecular Drug Susceptibility Test

This strategy involves identifying the mutations as resistance originates from genetic mutations. There are numerous mutations linked to resistance have been discovered, and molecular tests have been created to find them. Rapid turnaround times are one of the benefits of molecular drug susceptibility testing, but there are drawbacks as well, like as some compounds' limited sensitivity and a significant cost factor [34]. The common belief is that molecular assays must be carried out by specialized personnel. Certain tests, like the GeneXpert, are easy to use. It is possible to use a different test, known as the TruNat test, as a "near" point-of-care test away from a laboratory. Yet, up until recently, GeneXpert had a limited ability to detect resistance [34]. A novel molecular test called TrueNat can diagnose TB in under one hour and check for rifampicin resistance. The test's capabilities have been enhanced by several different assays.

Beacon Assays

Beacon assays employ ultra-sensitivity PCR to directly identify the *M. tuberculosis* complex and related rifampicin resistance from sputum samples. A closed tube system is one of the benefits of the GeneXpert TB assay, which is an automated real-time-based system with many other benefits. The GeneXpert TB test is recommended by the WHO; however, it has a few drawbacks [35].

Management of multi-drug resistance

The major goal of MDR-TB control is to stop it from developing in its initial place. This can be accomplished using the most economical MDR-TB therapy and prevention form, DOTS. However, because MDR-TB cases do not react well to SCC, it will be necessary to carefully introduce second-line medications to treat MDR-TB to stop the spread of these strains [4,36]. MDR-TB treatment aims to heal a particular patient and stop the spread of MDR-TB to others. In 2006, the WHO created recommendations for the systematic management of DR-TB, and in 2011, these guidelines were modified. These revised recommendations advocate for quick rifampicin resistance testing and a combination of four efficient medications comprising pyrazinamide, an injectable antibiotic, and a fluoroquinolone for treating individuals with MDR-TB [37]. The WHO recommended MDR-TB treatments with at least five efficient TB medications, comprising pyrazinamide and four second-line TB treatments, in the updated guidelines of 2016 shown in Table 2 [38].

**Table 2:** Classification of drugs for drug-resistant TB in 2016 Note: Updated from the World Health Organization's TB treatment suggestions TB: Tuberculosis Reference: [38]

Classification of drugs for drug-resistant TB in 2016		
Group	Classification	Medicine
A	Fluoroquinolones	Gatifloxacin
		Moxifloxacin
		Levofloxacin
B	Second-line injectable agents	Kanamycin
		Streptomycin
		Amikacin
		Capreomycin
C	Other core second-line agents	Linezolid
		Clofazimine
		Ethionamide or prothionamide
		Cycloserine or terizidone
D	Add-on agents D1	High-dose isoniazid
		Ethambutol
		Pyrazinamide
	D2	Delamanid
		Bedaquiline
	D3	Meropenem
		Amoxicillin/ clavulanate
		Para-aminosalicylic acid
		Imipenem/cilastatin

The medications used in the extended MDR-TB regimen were classified into three groups by the WHO Rapid Communication in 2018 (Table 3). Group A includes highly effective drugs such as bedaquiline, linezolid, and fluoroquinolones, strongly suggested for treating MDR-TB unless contraindicated. Clofazimine is part of group B and cycloserine or terizidone is a fallback option with certain limitations. Group C drugs can be considered if regimens with group A or B drugs are unsuccessful. Drugs in group C are categorized based on their benefits and drawbacks, with high-dose amoxicillin-clavulanate, kanamycin, isoniazid, and capreomycin being exceptions as they are not listed in this group [26,39].

**Table 3:** Classification of drugs for drug-resistant TB in 2019 TB: Tuberculosis Reference: [39]

Classification of drugs for TB that are resistant to many drugs in 2019		
Groups	Drugs	Steps
A	Moxifloxacin and Levofloxacin	Include each of the three medications (unless you cannot take them).
	Linezolid	
	Bedaquiline	
B	Cycloserine or terizidone	(Unless they can't be used) Add one or both medications.
	Clofazimine	
C	Amikacin or streptomycin	When medications from the two categories A and B are not utilized, add to a four- to five-drug regimen to complete it.
	Ethionamide or prothionamide	
	Imipenem cilastatin or meropenem	
	Pyrazinamide	
	Delamanid	
	Para amino salicylic acid	
	Ethambutol	

The information comes from a single set of WHO guidelines for treating TB that has become resistant to drugs [39].

Directly monitored treatment is crucial since MDR-TB development is typically linked to poor patient adherence, frequently accompanied by a doctor's subpar prescription. MDR-TB treatment plans should never be administered intermittently. Treatment for MDR-TB is linked to a protracted illness and impairment.

## Conclusions

MDR-TB is undeniably a significant universal health threat, and its emergence is an artificial problem. Various stakeholders, the public sector, the health care sector, medical professionals, and the sufferer's family, all bear some responsibility for contributing to this issue in their respective ways. The government's inadequate infrastructure and administrative controls on purchases without a robust quality control system within the National Tuberculosis Control Program contribute to the problem. The need for well-equipped laboratories further compounds the challenges in diagnosing and managing MDR-TB effectively. Medical professionals, particularly doctors, also play a role in the issue. Their lack of understanding about doses, therapy duration, side effects, typical regimens, treatment failures, and the emergence of medication resistance can be attributed to repeated brand name changes, insufficient patient motivation, and treatment failures. Patients and their families must also be aware of MDR-TB and the importance of adhering to their prescriptions diligently to decrease the risk of developing drug resistance. Raising awareness and eliminating the stigma associated with TB is crucial, as stigmatization or discrimination can hinder proper medical management and lead to missed opportunities for diagnosis, treatment interruptions, and unfavorable outcomes. Addressing the MDR-TB problem requires a comprehensive approach involving all stakeholders working together to improve infrastructure, enhance medical knowledge, ensure treatment adherence, and raise awareness to eliminate the stigma associated with TB. We can only effectively combat MDR-TB and reduce its impact on human health globally through collective efforts.
